# Recognition of facial expressions from minimal five-point-light displays

**DOI:** 10.3389/fpsyg.2026.1813559

**Published:** 2026-07-23

**Authors:** Sara Costa, Arianna Schiano Lomoriello, Elisa Straulino, Sonia Betti, Anita Ghislandi, Antonio Maffei, Fausto Caruana, Pier Francesco Ferrari, Luisa Sartori, Paola Sessa

**Affiliations:** 1 Department of Medicine and Surgery, Università di Parma, Parma, Italy; 2 Department of Developmental Psychology and Socialisation, University of Padova, Padova, Italy; 3 Padova Neuroscience Center (PNC), University of Padova, Padova, Italy; 4 Department of General Psychology, University of Padova, Padova, Italy; 5 Institute of Neuroscience, National Research Council of Italy (CNR), Parma, Italy; 6 Institut des Sciences Cognitives Marc Jeannerod, CNRS/Université Claude Bernard Lyon, Bron, France

**Keywords:** dynamic facial expressions, emotion recognition, facial action coding system (FACS), point-light displays, sensorimotor simulation

## Abstract

Facial expression recognition depends on both structural features (e.g., eye shape) and dynamic cues (e.g., muscle movements). However, the relative contribution of these information sources remains unclear, partly due to methodological challenges in isolating kinematic from configural information. Existing point-light display paradigms, while useful, typically employ numerous markers that still enable structural face recovery. Here, we introduce and validate the “Clepsydra” model—a minimal five-point configuration that captures the six basic emotions using markers on the eyebrows (×2), mouth corners (×2), and nose (×1). Stimuli derived from authentic facial expressions were presented to 91 participants in both point-light display (PLD) and full light (FL) conditions. Results showed above-chance recognition for five emotions in the PLD condition: happiness, surprise, anger, disgust, and sadness, while fear remained at chance level. Confusion matrix analyses revealed emotion-specific patterns, reflecting the diagnostic value of specific facial muscle movements (i.e., Action Units, AUs, as defined by the Facial Action Coding System): fear was primarily confused with surprise (sharing upper-face Action Units), while anger and disgust showed reciprocal misclassifications. These findings demonstrate that extremely sparse dynamic information can support emotion recognition, though with marked emotion-specific differences reflecting the diagnostic value of specific Action Units. The Clepsydra model offers a promising tool for isolating kinematic contributions to facial expression processing, with implications for testing sensorimotor simulation accounts and designing future minimal display paradigms.

## 1 Introduction

There is broad agreement among major models of face perception that our ability to decode facial information relies on processing both structural features—such as the overall configuration of the face—and dynamic cues, including facial muscle movements and gaze direction (Haxby et al., 2001; Bruce and Young, 1986; Pitcher and Ungerleider, 2021). Over the past decade, a growing number of researchers have proposed that interpreting these dynamic signals may involve sensorimotor simulation (Wood et al., 2016), a mechanism rooted in embodied cognition (Gallese and Sinigaglia, 2011), whereby motor and somatosensory regions involved in generating an action—such as moving a limb—are also recruited when observing the same action performed by someone else (Buccino et al., 2004; Rizzolatti and Craighero, 2004). The idea of an isomorphic relationship between action execution and action observation has likewise been extended to the domain of facial expressions (Del Vecchio et al., 2024; Ferrari et al., 2003; Iacoboni, 2009; Rayson et al., 2016; Wicker et al., 2003).

Several lines of evidence lend support to the sensorimotor simulation framework. Neuroimaging studies have demonstrated that simply observing facial expressions elicits activity in somatosensory and motor areas (Ensenberg et al., 2017; Ihme et al., 2014; Moore and Franz, 2017; Rayson et al., 2016; Sel et al., 2014; Wood et al., 2016), and importantly, that this activation appears to be selective for facial expressions rather than reflecting a general emotional response (Costa et al., 2025). Moreover, the phenomenon of facial mimicry—subtle, automatic, and rapid facial movements that mirror the emotional content of the observed expression—provides evidence of a direct matching mechanism between perceived expressions and the observer’s own motor representations (Dimberg et al., 2000; Hess and Blairy, 2001; Holland et al., 2021; Sato and Yoshikawa, 2007).

Yet despite this converging evidence, sensorimotor simulation remains a hypothesis rather than an established mechanism. While some lesion studies report impairments in expression recognition following damage to sensorimotor cortices (Adolphs et al., 2000; Pitcher et al., 2008), other research yields contradictory results (Japee et al., 2023; Schiano Lomoriello et al., 2024). A critical test case comes from individuals with Moebius syndrome—a condition causing lifelong facial paralysis. If sensorimotor simulation were necessary for recognition, these individuals should show marked deficits. However, they often perform comparably to controls on standard tasks (Rives Bogart and Matsumoto, 2010; Vannuscorps et al., 2020), suggesting reliance on the ventral visual pathway for structural analysis (Sessa et al., 2022). Importantly, deficits emerge when expressions are visually ambiguous, such as with morphed stimuli (Schiano Lomoriello et al., 2024) or highly degraded point-light displays (Vannuscorps and Caramazza, 2023).

This complex picture leads us to consider a critical yet often overlooked interpretive issue. Ventral routes, which primarily encode structural facial information, and sensorimotor simulation, supporting embodied recognition (Haxby et al., 2001), may contribute differentially depending on stimulus characteristics.

For instance, prototypical facial expressions could be recognized from structural information alone. In contrast, expressions that are either visually ambiguous or inherently dependent on kinematic features might rely more strongly on sensorimotor support for accurate decoding (Beffara et al., 2012; Karakale et al., 2024; Niedenthal et al., 2010; Wood et al., 2016). On this view, sensorimotor simulation constitutes not merely a downstream consequence of recognition, but a complementary perceptual strategy—one that becomes critical precisely when configural templates are underspecified. Testing this hypothesis requires stimuli that preserve kinematic information while degrading configural cues beyond what ventral-stream templates can support. Following Vannuscorps and Caramazza (2023), a point-light dataset of facial expressions appears to be ideally suited for this purpose: by stripping away surface features while preserving the spatiotemporal dynamics of movement, such stimuli selectively challenge configural processing and thus allow for a more direct test of the sensorimotor simulation hypothesis.

Several dynamic and ecologically valid databases of facial expressions have been developed, underscoring the importance of temporal and kinematic information in emotional processing (Zhang et al., 2014; Fernandes-Magalhaes et al., 2023). Attempts to capture this dynamic information have gone even further, with point-light facial expression stimuli also being developed (Bennetts et al., 2013; Bidet-Ildei et al., 2020; Decatoire et al., 2019; Takarae et al., 2021); while representing an important step toward isolating kinematic information, these typically employ numerous markers (e.g., 27 in Bennetts et al., 2013; 41 in Bidet-Ildei et al., 2020; Decatoire et al., 2019), or use incremental ranges that reach high densities (e.g., from 6 up to 54 markers in Takarae et al., 2021), allowing observers to infer underlying facial configuration.

What is currently missing, by contrast, is a *minimal* point-light model of facial expressions that preserves the essential dynamic signatures of basic emotions while strongly attenuating configurational information.

Here, we aim to address this gap by pushing point-light methodology to its minimal configuration: can healthy participants recognize the six basic emotions (happiness, sadness, anger, fear, disgust, and surprise) from just five facial markers? The “Clepsydra” model we adopt here places two points on the eyebrows, two at the corners of the mouth, and one on the nose (Straulino et al., 2023, 2025), representing an exceptionally sparse facial representation and the most minimal configuration within this class of models. This configuration is inherited from a dataset previously developed (Straulino et al., 2023, 2025), in which motion capture was applied to a minimal landmark layout designed to track the two facial regions most consistently associated with diagnostic kinematic information for basic emotions: the eyebrows (upper-face dynamics) and the mouth corners (lower-face dynamics), with a central nasal marker used as a positional anchor for normalization. These regions encompass the kinematic substrate of several AUs that are critical across multiple emotions (e.g., AU1 + 2 for surprise and fear, AU4 for anger, sadness and disgust, AU12 for happiness, AU15 for sadness; Ekman and Friesen, 1978). We therefore chose this configuration not because five markers represent a theoretical minimum, but because it is the most minimal layout available within an already-validated, motion-captured dataset of basic emotional expressions, and as such allows a principled test of how much emotion-relevant kinematic information survives at this extreme of sparsity.

This configuration is both minimal and uniform across all expressions, ensuring that differences in recognition performance reflect the intrinsic diagnostic value of motion in these regions rather than variation in marker coverage.

However, not all emotions rely equally on motion in the areas captured by these five points—some diagnostic Action Units (AUs) may fall entirely outside the tracked regions (Ekman and Friesen, 1978, 2003). This uniform approach thus allows us to systematically test which emotions remain recognizable when only this subset of facial kinematics is preserved, and whether recognition varies as a function of how well an emotion’s critical features are captured by the display.

Based on this rationale, we formulated both confirmatory and exploratory aims. Confirmatorily, we predicted that emotions whose diagnostic Action Units are captured by the tracked regions—particularly happiness (AU12), surprise (AU1 + 2), and anger (AU4)—would be recognized above chance from Clepsydra displays, whereas emotions relying on diagnostic cues falling outside, or only weakly represented within, the tracked regions would show recognition at or near chance level. Exploratorily, we examined the full structure of misclassifications across emotions, differences in confusion patterns between PLD and FL conditions, and the possible contribution of sensorimotor simulation under minimal-display conditions, as these analyses were not associated with specific point-prediction hypotheses.

Understanding which emotions survive extreme reduction will guide future model refinement and provide a methodological foundation for disentangling structural from dynamic contributions to facial expression processing, with direct implications for testing sensorimotor simulation accounts.

## 2 Materials and methods

### 2.1 Participants

A total of 91 participants (55 females, 36 males, mean age = 34.79; SD = 16.96) completed an online survey developed in Qualtrics^1^. The target sample size was determined with reference to previous studies employing comparable point-light display and facial expression recognition paradigms, which typically included smaller samples (e.g., *N* = 15–38; Bennetts et al., 2013; Bidet-Ildei et al., 2020; Takarae et al., 2021). Recruitment was conducted by distributing a QR code via social media platforms and through word-of-mouth. Participation was entirely voluntary and anonymous. All participants were native Italian speakers, and the questionnaire was administered in Italian. Prior to participation, all participants provided written informed consent before taking part in the study. The study was approved by the Ethics Committee of the University of Padova (Protocol no. 4842).

### 2.2 Stimuli creation and kinematic information

The video material used in the present study was originally created by Straulino et al. (2023, 2025). To generate the stimuli, a female actress (F) and a male actor (M) were recruited and instructed to imitate facial expressions corresponding to the six basic emotions: happiness, sadness, anger, surprise, fear, and disgust. Facial movements were recorded using a camera positioned above the monitor (Logitech C920 HD Pro Webcam, Full HD 1080p/30fps) and a SMART motion capture system (SMART-D, Bioengineering Technology and Systems [B|T|S]; 70 Hz sampling rate), with six infrared cameras arranged in a semicircle at a distance of 1–1.2 meters from the center of the room, which tracked reflective markers (5 mm in diameter) placed on specific facial areas—two above the eyebrows, one on the nose, and one at each corner of the mouth (i.e., Cheilions). The coordinates of the markers were then reconstructed with an accuracy of 0.2 mm in the field of view and analyzed with SMART-D Analyzer (kinematic data are reported in Table 1; for a detailed description of kinematic parameters see Straulino et al., 2025).

**Table 1:** 3D kinematic data for Full-Light video stimuli recorded by a female (F) actress and a male (M) actor.

Actors	Expression	MD CH	MD EB	MV CH	MV EB	MT CH	MT EB
F	Happiness	71.041	78.047	37.847	3.249	928.572	1014.286
M	Happiness	71.558	86.667	55.110	7.299	385.715	314.285
F	Surprise	57.605	78.930	0.165	9.327	514.285	357.142
M	Surprise	60.012	90.057	1.137	64.381	314.287	571.428
F	Fear	59.250	78.576	13.724	11.958	1142.858	1000.000
M	Fear	64.086	83.303	12.249	48.635	1114.286	1657.143
F	Sadness	58.023	78.110	10.858	52.517	857.145	1257.143
M	Sadness	62.501	85.312	0.544	0.235	1342.857	1342.412
F	Anger	60.114	77.985	20.862	4.113	1085.714	814.285
M	Anger	63.272	86.153	18.274	47.590	2228.572	3799.999
F	Disgust	58.533	79.142	13.450	14.113	928.571	1528.571
M	Disgust	62.101	84.913	0.313	0.044	785.714	800.000

Spatial parameters are: Maximum Distance of Cheilions (MD CH) and Maximum Distance of Eyebrows (MD EB). Velocity parameters are: Maximum Velocity of Cheilions (MV CH) and Maximum Velocity of Eyebrows (MV EB). Temporal parameters are: Movement Time of Cheilions (MT CH) and Movement Time of Eyebrows (MT EB).

In the present study, Point-Light Displays (PLD) and Full-Light Videos (FL) were extracted from that original material and two categories of stimuli were then produced: FL displaying the performer’s visible face expressing the emotion, and corresponding PLD showing only the markers’ movement as white dots on a black background, reproducing the same facial motion data. The videos were then edited using the iMovie software. Each clip consisted of three segments: a brief neutral phase (approximately 2-3 s), the target emotional expression (ranging between 3 and 6 s), and a short fade-out period (around 1 s). Stimuli were centered in the frame for clarity. For each emotion, both presentation formats and both identities were included, yielding a total of 24 videos (6 emotions × 2 formats × 2 identities).

### 2.3 Procedure

The experimental session began with an informed consent form and a description of the questionnaire. To ensure anonymity, participants were instructed to create a personal identification code based on their initials and the final two digits of their birth year. Subsequently, they completed a demographic questionnaire that included gender, biological sex, age, education level, and, when applicable, field of study. Participants indicated whether they currently practiced theater or regularly engaged in sports, as these activities may enhance sensorimotor skills relevant to perceiving and interpreting facial and bodily expressions (Conson et al., 2013; Rabini et al., 2024). Based on self-reports, 16 participants indicated practicing theater, 41 reported practicing sports, and 34 reported neither theatrical nor sports practice.

Before beginning the main task, participants completed a training block designed to familiarize them with the experimental procedure and the two stimulus formats. This block included five videos presented in both FL and PLD formats. After viewing each video, participants identified the emotion being expressed. Corrective feedback was provided following incorrect responses, whereas correct answers received brief affirmative feedback.

**Figure 1:**
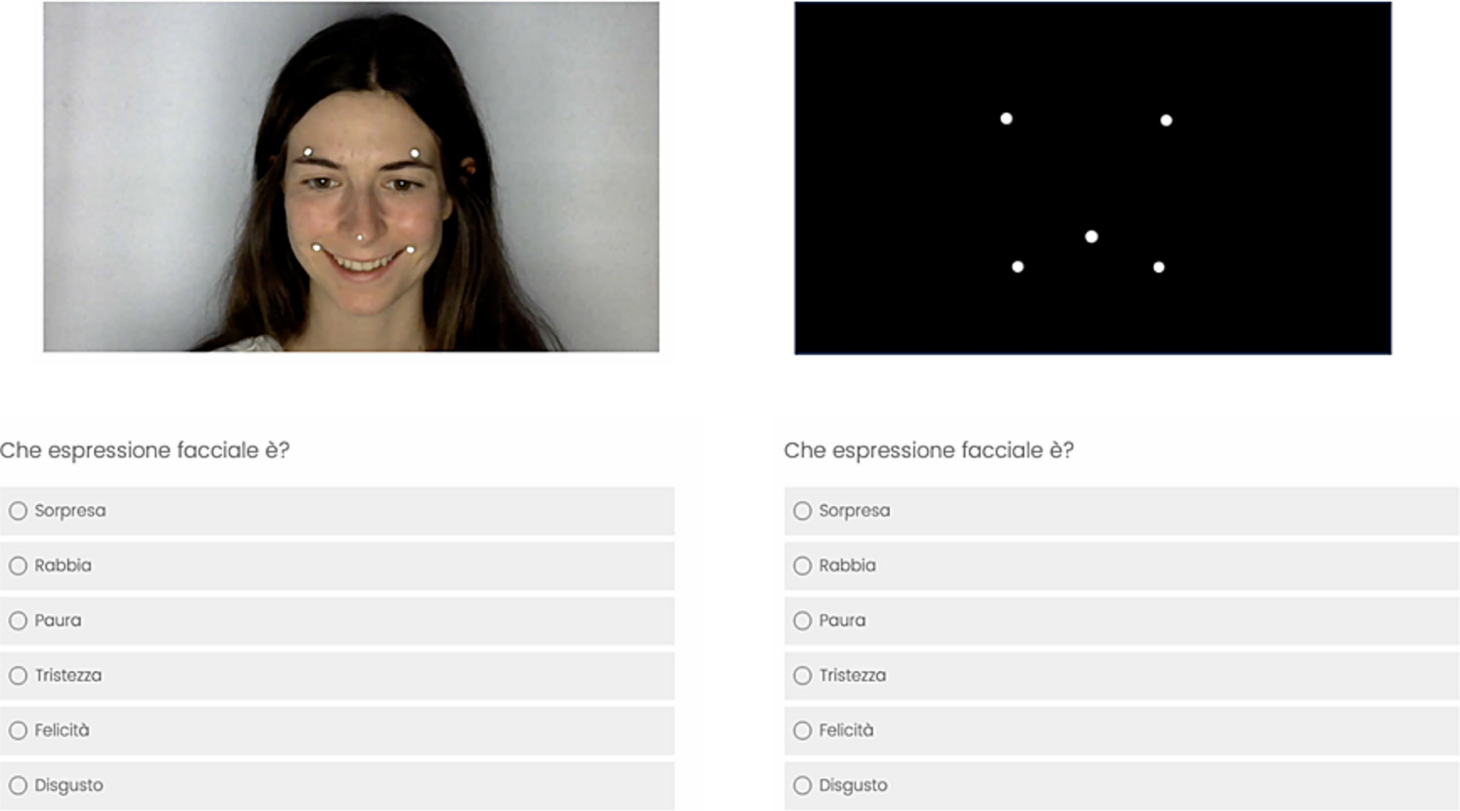
Example frames from the two stimulus conditions used in the experiment. On the left, the FL image displays the performer’s visible facial expression with reflective markers, whereas on the right, the PLD shows only the motion of the markers against a black background. In both conditions, participants were asked to identify which of the six basic emotions was portrayed.

The main experiment, following the training phase, consisted of two blocks presented without feedback. Each block comprised 48 randomized trials, corresponding to two presentations of each of the 24 unique stimuli, yielding a total of 96 trials for the entire session. During each trial, participants viewed a video and selected which of the six basic emotions was expressed (Figure 1).

### 2.4 Recognition strategy

To assess the strategies participants employed while interpreting the PLD, the survey included two additional questions. Midway through the experiment, participants responded to an open-ended question encouraging them to describe the strategy they were using. At the end of the study, they completed a multiple-choice item to indicate the primary strategy used during the task. The available options—presented in a random way across participants—included (a) simulating the observed movement, (b) attending to specific kinematic characteristics such as speed, rhythm, or amplitude, (c) relying on gut feeling or immediate impression, (d) excluding implausible options rather than directly identifying the emotion, (e) referring to the full-face videos as an anchor, (f) guessing without a clear strategy, or (g) selecting “Other” and specifying an alternative strategy in a free-response field.

### 2.5 Statistical analysis

All analyses were performed using the software R (version 2026.01.0) using the *glme*r function from the lme4 package (Bates et al., 2015). Prior to all analyses, trial-level responses were coded as correct (1) if the participant’s response matched the target emotion label, and incorrect (0) otherwise. Responses were compared against the emotion embedded in the stimulus identifier. For each participant × emotion × condition cell, the number of correct and incorrect responses were aggregated and entered into the models as a binomial outcome.

Recognition accuracy was analyzed using Generalized Linear Mixed Models (GLMMs) with a binomial distribution. Two linear mixed-effects models were fitted and compared using the Akaike Information Criterion (AIC). The first model included emotion as a fixed effect with only by-participant random intercepts, whereas the second model additionally included by-participant random slopes for emotion. The model with random slopes had a lower AIC (AIC = 2046.2) than the random-intercepts-only model (AIC = 2098.7), indicating a superior fit to the data and therefore was retained for the analyses. The chance level of accuracy was set to 1/6, corresponding to the probability of identifying the correct emotion through random guessing among the six available options. Estimated marginal means for each emotion were extracted from the fitted model using the emmeans package (Lenth et al., 2019). These means are expressed on the log-odds (logit) scale. The theoretical chance level (1/6) was converted to the same scale (log-odds = qlogis(1/6) = −1.609). For each emotion, the difference between the estimated marginal mean and the chance-level log-odds was computed, and a *z*-score was derived by dividing this difference by the standard error of the estimated marginal mean. All tests against chance were one-tailed, reflecting the directional hypothesis that recognition accuracy would exceed chance level, and *p*-values were adjusted using the Benjamini–Hochberg (BH) procedure to control the false discovery rate (FDR; Benjamini and Hochberg, 1995). Odds ratios relative to chance were computed by exponentiating the difference between each emotion’s estimated marginal mean (on the log-odds scale) and the log-odds corresponding to chance level (log-odds = qlogis(1/6) = −1.609). An odds ratio of 1.0 therefore indicates performance equivalent to chance, whereas values greater than 1.0 indicate above-chance recognition. Odds ratios were subsequently converted to Cohen’s *d* using the formula implemented in the effectsize package in R (Ben-Shachar et al., 2020).

Additional GLMM were fitted to test the interaction between sensorimotor experience (theater experience, sports practice, no structured activity) and format (PLD vs. FL) on recognition accuracy, with by-participant random intercepts. A separate model was also fitted to examine the interaction between observer sex and format (PLD vs. FL) as fixed effect and with and by-participant random intercepts, in order to assess whether recognition accuracy differed as a function of observer sex across stimulus sets.

Considering the differences in kinematic parameters between stimuli produced by the male and female performers (see Table 1), a further GLMM with a binomial distribution was fitted to examine whether recognition accuracy exceeded chance level separately for stimuli recorded by the male and the female performers. This model included a single fixed factor with 12 levels representing each emotion × performer combination, and by-participant random intercepts (fixed effects: emotion × performer; random effects: by-participant intercept). One-tailed tests against chance level (1/6) were then conducted for each level. All models used a binomial distribution with a logit link function and were fitted using the bobyqa optimizer.

To explore misclassification patterns in greater detail, confusion matrices were constructed and examined to identify tendencies in the misattribution of emotions. These matrices provided insight into which emotional expressions participants most frequently confused with one another.

Furthermore, recognition accuracy and confusion patterns were compared between the PLD and FL conditions in order to quantify the contribution of structural facial information to emotion-recognition performance. This comparison was conducted descriptively by examining the conditional response probabilities in the confusion matrices for each condition, and by comparing correct recognition rates across emotions between the two conditions.

To investigate systematic patterns of misclassification in PLD stimuli, a Bayesian multinomial mixed-effects model was fitted using the *bmrs* package. Indeed, a an accuracy-based measure relative to chance framework is insufficient to characterize confusion patterns when multiple incorrect response categories are chosen systematically above chance level—for instance, when participants respond “anger” to disgust stimuli more often than expected by chance, even if the correct response (“disgust”) remains the modal response. The multinomial model addressed this limitation by modeling the full distribution of response probabilities across all six emotions in the PLD categories simultaneously, as a function of the target emotion, with by-participant random intercepts. Following model fitting, posterior predictions were used to compute targeted contrasts between selected response categories for each target emotion—for example, comparing the posterior probability of a correct response against the posterior probability of a specific incorrect response. Contrasts were defined based on patterns observed in the confusion matrices and were therefore exploratory in nature. In this context, a Bayesian framework was specifically adopted because the contrasts were defined a posteriori based on observed confusion patterns, and because posterior credible intervals provide direct probability statements about the magnitude and direction of each contrast.

Exact model syntax is available in the analysis scripts deposited at https://osf.io/9d4ca.

## 3 Results

In the FL condition, recognition accuracy was significantly above chance for all six basic emotions (all *p*s < 0.001), providing a benchmark for expression recognition when full facial information is available. Effect sizes span from large to extremely large across emotions. Specifically, odds ratios relative to chance were 22.64 (*d* = 1.72) for Anger, 46.59 (*d* = 2.12) for Disgust, 3.97 (*d* = 0.76) for Fear, 835.11 (*d* = 3.71) for Happiness, 15.14 (*d* = 1.50) for Sadness, and 126.22 (*d* = 2.67) for Surprise.

**Figure 2:**
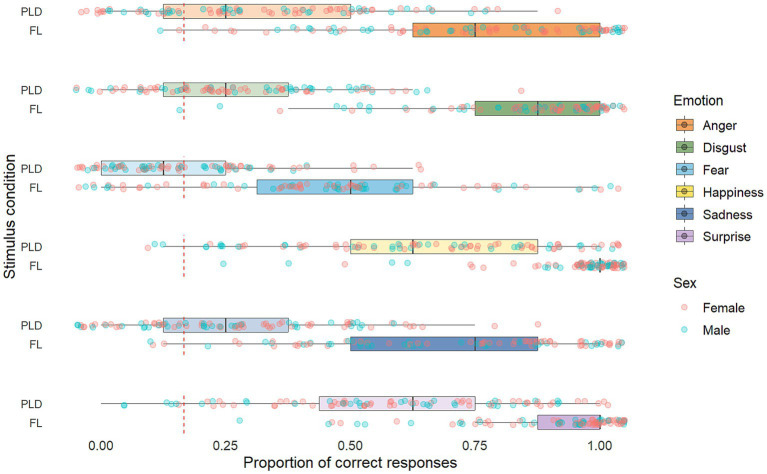
Boxplots depict the proportion of correct responses across participants for each emotion and stimulus condition (e.g., the probability of responding Anger when anger is the presented stimulus). The dashed red vertical line marks chance-level performance (1/6). Individual dots represent participants’ accuracy scores, color-coded by sex. Colors denote emotion categories, with darker shades indicating FL and lighter shades indicating PLD. For Happiness in the FL condition, performance approached ceiling levels, resulting in visually compressed distributions near the upper bound of the scale.

Having established this baseline, we then examined recognition performance in the PLD condition. Recognition accuracy was significantly above chance after FDR correction for Anger (*z* = 6.87, *p* < 0.001, OR = 2.23, *d* = 0.44), Disgust (*z* = 6.17, *p* < 0.001, OR = 1.84, *d* = 0.34), Happiness (*z* = 16.58, *p* < 0.001, OR = 10.02, *d* = 1.27), Sadness (*z* = 4.31, *p* < 0.001, OR = 1.68, *d* = 0.28), and Surprise (*z* = 16.34, *p* < 0.001, OR = 7.45, *d* = 1.11). Recognition accuracy for Fear did not differ from chance (*z* = −0.16, *p* = 0.43) (Figure 2).

A GLMM examining the effects of the format (PLD vs. FL), sensorimotor experience group (theater experience, sports practice, no activity), and their interaction revealed a significant main effect of format (*χ*^2^ (1) = 194.53, *p* < 0.001), indicating higher recognition accuracy in the FL condition compared to the PLD condition. The main effect of the group was not significant (*χ*^2^ (2) = 0.35, *p* = 0.84), suggesting no overall differences in accuracy between theater-experienced, sports-practice, and control participants. Importantly, the group × format interaction was also not significant (*χ*^2^ (2) = 2.49, *p* = 0.29), indicating that the effect of the format did not differ across sensorimotor experience groups. Observer sex was also included as a fixed effect; however, no significant main effect of the observer sex was observed (*χ*^2^ (1) = 3.40, *p* = 0.065), nor did the observer sex interact with format (*χ*^2^ (1) = 0.64, *p* = 0.423), indicating comparable recognition accuracy between males and females participants across conditions.

Finally, a GLMM was conducted to examine performance above chance level separately for stimuli produced by the two performers (Table 1). After FDR correction, Happiness and Surprise showed significant above-chance recognition for both performers, with larger effects for the male performer (happiness: OR = 15.45, *d* = 1.51; surprise: OR = 9.22, *d* = 1.23) than for the female (happiness: OR = 5.60, *d* = 0.95; surprise: OR = 5.54, *d* = 0.94). Anger and Disgust were also significant for both performers but with more moderate effect sizes, again larger for the male (anger: OR = 3.25, *d* = 0.65; disgust: OR = 2.35, *d* = 0.47) than for the female performer (anger: OR = 1.67, *d* = 0.28; disgust: OR = 1.40, *d* = 0.19). Sadness reached significance only for the male performer (OR = 2.88, *d* = 0.58), while Fear was not significant for either performer (*p*s = 0.182).

In sum, observers could recognize most emotions from minimal kinematic information alone, though with reduced accuracy compared to FL videos. Recognition performance did not vary as a function of sensorimotor expertise, though the interpretation of this null effect remains open. Although overall differences between stimuli produced by the two performers were not substantial, the larger effect sizes observed for the male performer—occasionally resulting in meaningful differences (e.g., for sadness)—suggest that variations in the spatial amplitude and velocity of facial movements may contribute to differences in recognition performance.

**Figure 3:**
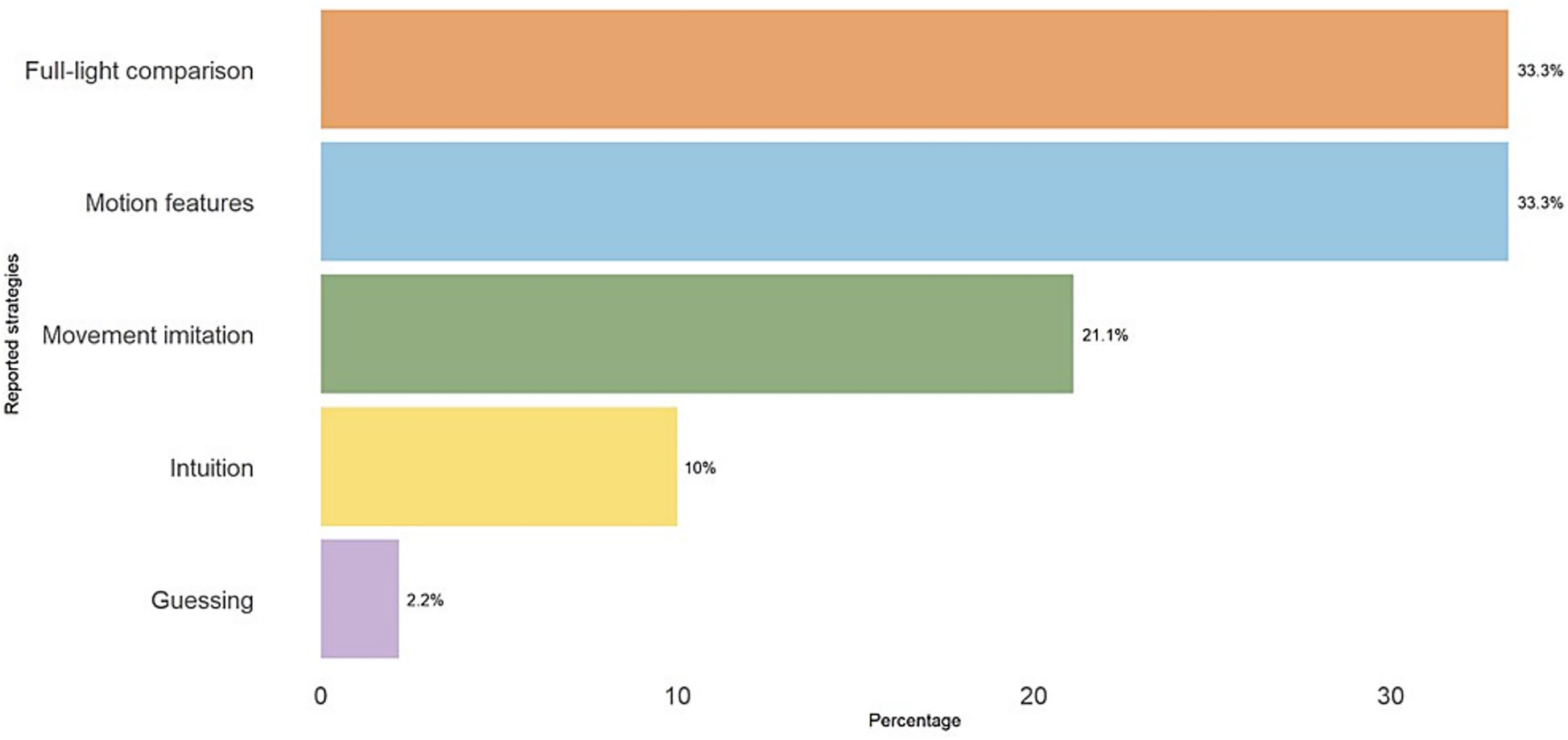
Percentage distribution of reported recognition strategies.

Descriptive analyses of reported strategies indicated that comparison with the full-light condition and reliance on movement features were the most frequent (33.3%), followed by movement imitation (21%), with gut feeling/impression (10%) and guessing (2.2%) occurring least frequently (Figure 3).

Then, at the explicit, self-reported level, participants described actively engaging with the available motion information, predominantly relying on kinematic analysis and on comparisons of the stimuli with the full light model, rather than unsystematic guessing.

**Figure 4:**
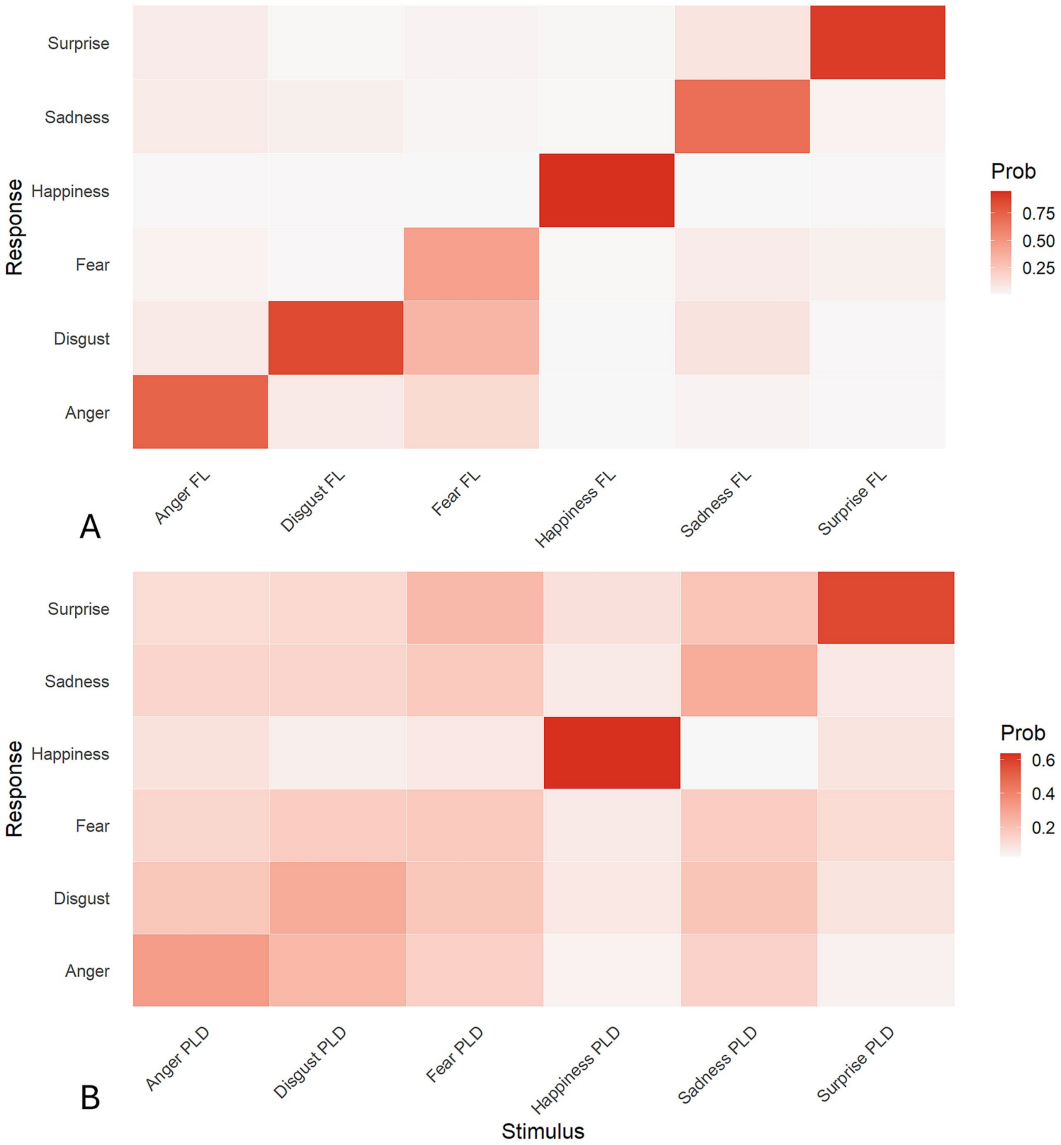
The heatmap shows conditional probabilities of participants’ responses (rows) given the presented emotional stimulus (columns) for the FL stimuli **(A)** and PLD **(B)**. Color intensity reflects the probability of each response, with darker shades indicating higher values. Correct identifications are shown along the diagonal.

Confusion matrix analyses were then performed to examine patterns of emotion recognition and misclassification in the FL and PLD conditions, showing remarkably different patterns. In the FL, Fear was most frequently misidentified as Disgust (34.5%) and, to a lesser extent, as Anger (15.0%) and Sadness (6.3%). Anger responses showed a tendency to be confused with Fear (15.0%), whereas Sadness was occasionally misclassified as Surprise (10.3%) (Table 2; Figure 4A). As expected, the PLD condition showed a marked dispersion of responses (Table 3; Figure 4B). Fear exhibited the greatest perceptual ambiguity, with responses distributed across multiple categories, most notably Surprise (23.5%), Sadness (17.6%), Disgust (16.6%), and Anger (15.6%). Similarly, Anger and Disgust stimuli showed reciprocal confusions, while Sadness responses were frequently misattributed to other negative emotions. Despite this overall increase in confusion, Happiness and Surprise retained a relatively selective response profile.

**Table 2:** The table reports conditional probabilities of each response (rows) given the presented emotional stimulus (columns) in the FL condition.

Response	Stimulus					
	Anger FL	Disgust FL	Fear FL	Happiness FL	Sadness FL	Surprise FL
Anger	**0.75**	0.07	0.15	0.00	0.03	0.07
Disgust	0.08	**0.85**	0.34	0.00	0.10	0.07
Fear	0.03	0.01	**0.45**	0.01	0.06	0.04
Happiness	0.01	0.01	0.00	**0.95**	0.00	0.01
Sadness	0.07	0.04	0.02	0.01	**0.70**	0.03
Surprise	0.07	0.01	0.02	0.02	0.10	**0.91**

Values represent the proportion of responses assigned to each emotion for a given stimulus. The bold values represent the proportion of correct responses (e.g., the answer ‘Anger’ when Anger was the presented emotion).

**Table 3:** The table reports conditional probabilities of each response (rows) given the presented emotional stimulus (columns) in the PLD condition.

Response	Stimulus					
	Anger PDL	Disgust PDL	Fear PDL	Happiness PDL	Sadness PDL	Surprise PDL
Anger	**0.32**	0.24	0.15	0.04	0.14	0.04
Disgust	0.19	**0.28**	0.18	0.08	0.19	0.09
Fear	0.13	0.17	**0.17**	0.07	0.17	0.11
Happiness	0.1	0.06	0.07	**0.63**	0.03	0.09
Sadness	0.14	0.14	0.17	0.07	**0.27**	0.07
Surprise	0.11	0.12	0.23	0.10	0.20	**0.58**

Values represent the proportion of responses assigned to each emotion for a given stimulus. The bold values represent the proportion of correct responses (e.g., the answer ‘Anger’ when Anger was the presented emotion).

Overall, the transition from FL to PLD led to a general increase in perceptual ambiguity, yet this effect was not uniform: while negative emotions became largely interchangeable, Happiness and Surprise maintained relatively distinct perceptual profiles, pointing to differential reliance on configural versus kinematic cues across emotion categories.

Based on the confusion matrix and Supplementary Figure S1, some incorrect response categories were chosen more frequently than expected by the chance level (16.7%), warranting examination beyond binary accuracy (see Statistical Analysis section for details). Results of the Bayesian multinomial mixed-effects model revealed the following patterns. For Fear stimuli, participants showed substantial confusion between emotion categories, with Fear and Anger responses largely indistinguishable (mean Δ = 0.03, 95% CI [−0.03, 0.08]). Similarly, the difference between Disgust and Fear responses was uncertain (mean Δ = −0.02, 95% CI [−0.07, 0.03]). However, Surprise responses were more frequent than Fear responses (mean Δ = −0.06, 95% CI [−0.13, −0.01]). For Disgust stimuli, while Disgust responses tended to be more frequent than Anger responses, showing directional evidence, the 95% credible interval marginally overlapped zero (mean Δ = 0.06, 95% CI [−0.01, 0.12]). For Sadness stimuli, participants favored Sadness over Surprise responses, with a positive mean difference and a credible interval excluding zero (mean Δ = 0.08, 95% CI [0.03, 0.15]). For Anger stimuli, Anger responses were markedly more frequent than Disgust responses (*p* (Anger > Disgust) = 1.00; mean Δ = 0.15, 95% CI [0.09, 0.22]).

Together, these findings reveal a structured pattern of confusions: Fear lacked a distinct perceptual identity and was systematically assimilated to other negative emotions, whereas Anger, Sadness, and Disgust—despite reciprocal confusions—retained measurable categorical boundaries. This asymmetry suggests that the kinematic signatures of some emotions are more resilient to the loss of configural information than others—a pattern that may reflect which action units are preserved versus lost in the minimal marker configuration of the Clepsydra model (see Discussion).

## 4 Discussion

The present study investigated whether facial expressions can be accurately recognized when structural information is drastically reduced, leaving only sparse kinematic cues. Using a minimal five-point-light model, we aimed to isolate the dynamic component of facial expressions (Krumhuber et al., 2023) and assess whether motion information alone can support emotion recognition.

Results indicate that observers recognize several basic emotions from minimal PLD at above-chance levels, confirming that while structural information provides a strong advantage, dynamic cues alone can carry diagnostically relevant emotional information. Crucially, recognition performance varied substantially across emotions. Happiness and Surprise showed relatively robust recognition even without configural cues, while Fear exhibited pronounced ambiguity, with responses distributed across multiple emotion categories.

The classical Facial Action Coding System (FACS; Ekman and Friesen, 1978; Bartlett et al., 1995) could offer a useful framework for interpreting these patterns. According to this framework, each basic emotion is defined by a constellation of diagnostic AUs (Action Units, Ekman and Friesen, 1978), only some of which were captured by the Clepsydra configuration (see Supplementary Table S1). This asymmetry in AU coverage maps systematically onto recognition performance.

Happiness achieved the highest PLD accuracy (63%), likely because its most diagnostic cueAU12 (lip corner puller; Ekman and Friesen, 1978) was directly captured by the mouth markers. AU12 is both necessary and sufficient for the recognition of a posed smile (Ruan et al., 2020; Calvo and Nummenmaa, 2016; Ekman et al., 1990) and crucially, its biomechanical action is fully preserved in the five-point display: the lateral displacement of the lip corners is entirely captured by the mouth markers, leaving the defining movement of happiness with no information loss relative to the full-light condition. Surprise similarly retained robust recognition (58%), plausibly because the combination of raised eyebrows (AU1 + 2) (Izard, 1979; Ekman and Friesen, 1978) and downward mouth movement (partially indexing AU26; Ekman and Friesen, 2003) produces a distinctive kinematic signature even with sparse markers. By contrast, Fear yielded chance-level performance and maximal response dispersion. This pattern is theoretically predictable. Fear and surprise share identical upper-face action units (AU1 + 2; Izard, 1979; Ekman and Friesen, 1978), and their critical distinguishing features were largely absent from the minimal display. The AU20 (lip stretcher; Izard, 1979; Ekman and Friesen, 1978), which contributes to the tension of the lower face, was not captured by the five-point configuration. The Bayesian analysis confirmed that Fear and Surprise responses were statistically indistinguishable for Fear stimuli, consistent with the loss of the disambiguating cue. The reciprocal confusion between Anger and Disgust follows analogous logic. Both involve brow lowering (AU4), which was captured. However, their distinguishing features–AU9 (nose wrinkle) for Disgust (Hjortsjö, 1970; Izard, 1979; Ekman and Friesen, 1978) and AU23/24 (lip tightening; Ekman and Friesen, 1978) for Anger–involve either surface deformation or subtle lip tension that the 5-point model cannot represent. The nose marker tracks position, not wrinkling; the mouth markers track corner displacement, not pressing or tightening.

Sadness showed moderate accuracy (27%) with confusion primarily toward other negative emotions (Anger, Disgust, Fear). While AU1, AU4, and AU15 were theoretically captured, the kinematic excursion of sadness expressions is typically subtle–small, slow movements that may not generate a distinctive dynamic signature when reduced to five points. In this light, it is unsurprising that above-chance recognition emerged only for the male performer, whose expressions were characterized by slower and more prolonged movements (Table 1). This kinematic profile is arguably more naturalistic and canonical for sadness, which is associated with low-intensity, temporally extended muscle activity (Ekman and Friesen, 2003, 1978), and may have provided a richer and more informative dynamic signal even within the constraints of the minimal display.

In short, recognition accuracy in the minimal display was largely predictable from AU coverage: emotions whose diagnostic features fell within the marker locations remained identifiable, whereas those relying on surface deformation, subtle muscle tension, or regions outside the tracked points lost their perceptual distinctiveness.

Comparing PLD and FL accuracy reveals substantial performance drops for all emotions (Table 2). However, interpreting these differences requires caution. The PLD condition differs from the FL not only is configurational facial information removed, but parts of the kinematic signal are also selectively lost or undersampled due to the minimal number of tracked points. For tracked regions, the model captures *positional kinematics* (landmark displacement over time) but not *surface kinematics* (skin wrinkling, muscle tensioning, textural changes that occur with minimal spatial excursion). AU9 (nose wrinkler), for instance, involves contraction that produces visible skin folds, yet the nose itself barely moves in space–a single positional marker cannot capture this.

These distinctions have direct implications for interpreting the emotion-specific patterns. Happiness and Surprise showed the smallest performance drops and remained well above chance, suggesting that their core kinematic signatures—mouth corner displacement (AU12) and brow raising (AU1 + 2)—are well captured by the Clepsydra configuration.

Disgust and Anger showed large drops, but for partially different reasons. Disgust relies heavily on AU9, a surface deformation that no positional point-light model can capture. Anger’s diagnostic features include both untracked movements (AU5, AU7: eyelid) and surface tension (AU23/24: lip pressing)—information lost for distinct reasons.

Fear presents a particularly instructive case. Already poorly recognized in the FL condition (45%), it fell to chance in the PLD condition. Critically, Fear’s diagnostic cues–AU5 (upper lid raiser) and AU20 (lip stretcher)—are *movements*, not structural features. They are absent from the PLD not because kinematics are inherently insufficient for Fear, but probably because the Clepsydra model does not track the relevant regions.

Thus, the present data support a nuanced conclusion: the five-point Clepsydra configuration preserves sufficient positional kinematics to support above-chance recognition for five of six basic emotions. For Fear, and partially for Disgust, recognition failure may reflect either (a) genuine dependence on configural information, (b) loss of diagnostic movement from untracked regions, or (c) reliance on surface kinematics that positional tracking cannot capture. Disambiguating these possibilities would require comparing the Clepsydra model to PLD configurations that track eyelid, lip, and cheek movement.

Regarding the strategies reported by participants, results revealed three distinct approaches. A first group (33.3%) attempted to reconstruct the configural aspects of the face, trying to retrieve the full facial percept from the sparse display. A second group (33.3%) abandoned this strategy in favor of focusing on the kinematic characteristics of the motion, while a third group (21.1%) engaged in imitative behavior—reproducing the movements to access their motor programs. Taken together, the majority of participants (54.4%) thus relied on motion—or simulation-based (Wood et al., 2016) strategies rather than configural reconstruction, suggesting that minimal PLD stimuli engage sensorimotor processing and that this paradigm may be particularly well suited for dissociating configural from dynamic and simulation-based routes to facial emotion recognition.

### 4.1 Limits and future directions

A limitation of the study concerns the theoretical rationale for the specific five-marker configuration adopted here. As stated in the Introduction section, the configuration was inherited from the previously validated dataset of Straulino et al. (2023, 2025), in which markers were placed on the facial regions most consistently associated with diagnostic kinematic information for the six basic emotions (eyebrows, mouth corners), plus a nasal positional anchor. Although this layout maps onto the locations of several AUs critical across multiple expressions (notably AU1 + 2, AU4, AU12, AU15), the present design does not formally establish that five markers represent the empirical minimal threshold for emotion recognition, nor does it characterize the marginal contribution of each individual marker.

Building on these findings, future research may extend this approach in two directions. First, a data-driven strategy based on eye-tracking could be employed to inform marker placement. By recording observers’ gaze patterns during facial expression recognition tasks, it would be possible to identify the facial regions preferentially sampled when decoding specific emotions, yielding sparse configurations that reflect empirically derived perceptual priorities rather than fixed anatomical assumptions. Second, minimal point-light models could be designed on an emotion-specific basis, with marker placement tailored to the most diagnostic action units for each expression. Rather than adopting a uniform configuration, different sparse models could selectively preserve the motion of critical AUs—such as eyelid movement for fear, or lip pressing for anger—allowing a direct test of whether restoring the kinematics of specific action units is sufficient to recover recognition performance. Together, these approaches would enable the construction of minimal yet functionally optimized point-light datasets, providing a powerful framework for probing dynamic information underlying facial emotion recognition.

A limitation of the study concerns the number of encoders. Only two actors were employed, a choice driven by two practical considerations: the proof-of-concept nature of the study, which prioritized tight control over the kinematic signal rather than broad sampling across encoders, and the length of the experiment, which precluded adding further identities without substantially increasing participant fatigue in an online setting. Notably, the kinematic parameters reported in Table 1 (Maximum Distance, Maximum Velocity, and Movement Time of Cheilions and Eyebrows), which were extracted from the original motion-capture recordings (Straulino et al., 2023, 2025), revealed largely consistent expressive patterns across the two performers, with meaningful differences emerging only for sadness, suggesting that the observed effects are unlikely to reflect idiosyncratic features of a single encoder. Nevertheless, the present design does not permit conclusions about the generalizability of these findings across a wider range of encoders, and future studies would benefit from including a larger and more diverse set of actors.

A further limitation concerns possible memory-related effects. Each unique stimulus was presented four times over the course of the session, and a substantial proportion of participants spontaneously reported anchoring the PLD percept to the FL version of the same stimulus. Both factors could in principle contribute to recognition accuracy in the PLD condition independently of the kinematic signal itself. Several design features limit this concern—within-block randomization and the absence of performance feedback reduced opportunities for trial-by-trial learning—and an additional analysis found no evidence of systematic accuracy improvement across blocks (see Supplementary material). We acknowledge, nonetheless, that the specific strategy of anchoring the PLD percept to a previously seen FL version cannot be fully ruled out, and future designs should employ fully independent stimulus sets to address this caveat directly.

Regarding the comparison among groups (actors vs. sport practitioners vs. controls), no significant group difference emerged, a null result that is difficult to interpret given the unbalanced sample size, with the absence of professional training. Future studies recruiting larger and more carefully selected samples—spanning a broader range of expressive training, from naïve observers to professional actors—would allow a more powerful and systematic test of whether expertise in facial motor production modulates the reliance on simulation-based strategies and, in turn, recognition performance under minimal PLD conditions.

Beyond methodological refinement, these findings carry broader implications for understanding the neural architecture of facial expression processing. Current evidence suggests that ventral visual pathways and sensorimotor simulation mechanisms contribute differentially to emotion recognition depending on stimulus characteristics (Haxby et al., 2001; Wood et al., 2016), and minimal PLD conditions—by removing structural information—may place particular demands on the latter. A first indication of this comes from the strategy data collected in the present study: a substantial proportion of participants reported relying primarily on kinematic cues, with some explicitly describing imitative behavior as their recognition strategy. This spontaneous recruitment of sensorimotor processing suggests that minimal PLD stimuli may be well suited for testing clinical populations in whom such mechanisms are compromised. Specifically, if sensorimotor simulation serves as a compensatory route when structural information is unavailable, individuals with impaired motor or somatosensory systems should show disproportionate recognition deficits under minimal PLD conditions while maintaining relatively preserved performance with full-light stimuli.

## 5 Conclusion

These findings demonstrate that minimal point-light displays can support facial expression recognition, albeit with substantial emotion-specific variation. While representing an initial proof-of-concept, this work establishes the feasibility of using extremely sparse configurations to isolate dynamic information in facial expression processing. The pattern of successes and failures across emotions provides a roadmap for systematic refinement: by revealing which kinematic features are preserved and which are lost, the Clepsydra model highlights the critical role of marker placement in capturing diagnostically relevant movement. Moving forward, integrating empirical data from eye-tracking, confusion patterns, and action unit analysis offers a principled approach to designing optimized minimal displays. Such tools hold promise not only for advancing theoretical models of emotion recognition but also for providing ecologically valid yet experimentally controlled stimuli to test the boundaries of sensorimotor simulation in face perception.

## Data Availability

Publicly available datasets were analyzed in this study. The data are available on the Open Science Framework (OSF) at https://osf.io/9d4ca.

## Footnotes

1
https://www.qualtrics.com
