# Supplementary material for: Recognition of facial expressions from minimal five-point-light displays
Source: Front Psychol. 2026 Jul 23;17:1813559. doi: 10.3389/fpsyg.2026.1813559 (PMC13441706; doi:10.3389/fpsyg.2026.1813559)
Supplement: Supplementary file 1 [file Data_Sheet_1.DOCX]

## **SUPPLEMENTARY MATERIALS**

**Supplementary Analyses**

**Learning effects across presentation blocks**

Given the four repetitions of each stimulus across the experiment, we assessed potential learning effects by examining performance across the two experimental blocks. Specifically, the first and second presentations of each stimulus occurred within the first block (prior to the strategy question), while the third and fourth presentations occurred within the second block. This design allowed us to test whether recognition accuracy for PLDs improved between the first and second half of the experiment. To this end, we fitted a GLMM including emotion, block (first vs. second presentation half), and their interaction as fixed effects, with by-participant random intercepts. Neither the main effect of block (χ²(1) = 0.007, p = .936) nor the block × emotion interaction (χ²(5) = 8.56, p = .128) reached statistical significance, indicating that recognition accuracy did not systematically vary across blocks and that the pattern of responses across emotion categories remained stable throughout the task.

**Observe sex × performer sex interaction**

To test whether the effect of performer sex on recognition accuracy interacted with observer sex — as might be predicted under a simulation-based account — we fitted an additional mixed-effects binomial regression model including observer sex, performer sex, and their interaction as fixed effects, with by-participant random intercepts. The analysis revealed a significant main effect of performer sex (χ²(1) = 47.39, p < .001), whereas neither the main effect of observer sex (χ²(1) = 0.76, p = .383) nor the observer sex × performer sex interaction (χ²(1) = 0.25, p = .621) reached significance. These results indicate that, although recognition performance differed as a function of performer sex, no evidence was found for a same-sex advantage in emotion recognition.

Fig. S1. Response probability distributions by target emotion. Each horizontal bar represents a target emotion presented as stimulus, with colored segments indicating the proportion of responses assigned to each emotion category. Probabilities are normalized within target emotions, allowing comparison of response patterns and misclassification tendencies.

Table S1. Diagnostic Action Units for each basic emotion and their detectability in the Clepsydra model. Note. ✓ = captured; ✗ = not captured; ~ = partially inferable from marker displacement.

| **Emotion** | **Diagnostic AUs** | **AU Description** | **Captured by Clepsydra?** |
| --- | --- | --- | --- |
| **Happiness** | AU6 | Cheek raiser | ✗ |
|  | AU12 | Lip corner puller | ✓ (mouth markers) |
| **Sadness** | AU1 | Inner brow raiser | ✓ (eyebrow markers) |
|  | AU4 | Brow lowerer | ✓ (eyebrow markers) |
|  | AU15 | Lip corner depressor | ✓ (mouth markers) |
| **Anger** | AU4 | Brow lowerer | ✓ (eyebrow markers) |
|  | AU5 | Upper lid raiser | ✗ |
|  | AU7 | Lid tightener | ✗ |
|  | AU23/24 | Lip tightener/presser | ✗ |
| **Fear** | AU1+2 | Inner + outer brow raiser | ✓ (eyebrow markers) |
|  | AU4 | Brow lowerer | ✓ (eyebrow markers) |
|  | AU5 | Upper lid raiser | ✗ |
|  | AU20 | Lip stretcher | ✗ |
|  | AU26 | Jaw drop | ~ (partially inferable) |
| **Disgust** | AU9 | Nose wrinkler | ✗ |
|  | AU10 | Upper lip raiser | ✗ |
|  | AU15 | Lip corner depressor | ✓ (mouth markers) |
| **Surprise** | AU1+2 | Inner + outer brow raiser | ✓ (eyebrow markers) |
|  | AU5 | Upper lid raiser | ✗ |
|  | AU26 | Jaw drop | ~ (partially inferable) |

Table S2. Recognition accuracy (%) in Full-Light and PLD conditions.

| **Emotion** | **FL** | **PLD** | **Δ** | **Primary information lost in PLD** |
| --- | --- | --- | --- | --- |
|  |  |  |  |  |
| Happiness | 95% | 63% | −32% | AU6 (surface: cheek raising) |
| Surprise | 91% | 58% | −33% | AU5 (untracked: eyelid raising) |
| Disgust | 85% | 28% | −57% | AU9 (surface: nose wrinkling), AU10 (untracked: levator labiis) |
| Anger | 75% | 32% | −43% | AU5, AU7, AU23 (untracked + surface) |
| Sadness | 70% | 27% | −43% | Low-amplitude movement |
| Fear | 45% | 17% | −28% | AU5, AU20 (untracked: eyelid, lip stretch) |
